# Can animal data translate to innovations necessary for a new era of patient-centred and individualised healthcare? Bias in preclinical animal research

**DOI:** 10.1186/s12910-015-0043-7

**Published:** 2015-07-28

**Authors:** Susan Bridgwood Green

**Affiliations:** SABRE Research UK, PO BOX 18653, London, NW3 4UJ UK

**Keywords:** Systematic review, Bias, Animal models, Research volunteers, Patients, Pre-clinical, Translational, Reproducibility, Waste in research, Phase 1 trials

## Abstract

**Background:**

The public and healthcare workers have a high expectation of animal research which they perceive as necessary to predict the safety and efficacy of drugs before testing in clinical trials. However, the expectation is not always realised and there is evidence that the research often fails to stand up to scientific scrutiny and its 'predictive value' is either weak or absent.

**Discussion:**

Problems with the use of animals as models of humans arise from a variety of biases and systemic failures including: 1) bias and poor practice in research methodology and data analysis; 2) lack of transparency in scientific assessment and regulation of the research; 3) long-term denial of weaknesses in cross-species translation; 4) profit-driven motives overriding patient interests; 5) lack of accountability of expenditure on animal research; 6) reductionist-materialism in science which tends to dictate scientific inquiry and control the direction of funding in biomedical research.

**Summary:**

Bias in animal research needs to be addressed before medical research and healthcare decision-making can be more evidence-based. Research funding may be misdirected on studying 'disease mechanisms' in animals that cannot be replicated outside tightly controlled laboratory conditions, and without sufficient critical evaluation animal research may divert attention away from avenues of research that hold promise for human health. The potential for harm to patients and trial volunteers from reliance on biased animal data^1^ requires measures to improve its conduct, regulation and analysis. This article draws attention to a few of the many forms of bias in animal research that have come to light in the last decade and offers a strategy incorporating ten recommendations stated at the end of each section on bias. The proposals need development through open debate and subsequent rigorous implementation so that reviewers may determine the value of animal research to human health. The 10Rs + are protected by a Creative Commons Attribution 3.0 Unported License and therefore may be 'shared, remixed or built on, even commercially, so long as attributed by giving appropriate credit with a link to the license, and indicate if changes were made.’

**Electronic supplementary material:**

The online version of this article (doi:10.1186/s12910-015-0043-7) contains supplementary material, which is available to authorized users.

## Introduction

The concept of using animals as surrogate models for humans relies on the conjecture that animal species predict the human outcome. However, bias and conflict of interest make it difficult to confirm the hypothesis and evidence suggests that animal studies are inconsistent in translation to human health; [1, 2] rather than delivering reliable answers to research questions they are often overinterpreted [3].

While there remains a lack of scientific robustness in animal research and failure to sufficiently screen for poor quality research, this situation will negatively impact on our healthcare system. It might then be unreasonable to expect continued public and professional support for animal research if reviewers are unable to determine either the scientific or ethical quality of the studies.

## Discussion

Various forms of bias are found throughout animal research; in the design, reporting, publication, analysis, regulation, funding and validation of animal models. Failure to adequately correct for bias by utilising the best available research methods is evidenced by misleading results [4], which have needlessly involved thousands of participants in clinical trials [5–9] and wasted funding and resources [10].

In 2004 the US FDA produced a white paper that looked at the disconnect between increased expenditure in R&D and the high attrition (drop-out) rate in drug discovery [11]. The report was critical of pre-clinical animal models and called for better models or better ways of predicting the outcome of drug trials in patients. A decade later, in spite of extensive funding for the development and improvement of animal models, [12, 13] the attrition rate has not fallen and it is estimated that the likelihood of a drug successfully passing Phase III is 50 %, and that the overall attrition rate of late-stage drug development is unsustainable [14]. Only 11 % of medical compounds are approved for use following years of research and development [15, 16].

The Lancet published a review in 2009 of the production and reporting of biomedical research which calculated 85 % of basic and clinical research is wasted because of inadequate or inappropriate design, non-publication and poor reporting [17] resulting in an estimated global annual loss of over $100b research funding [18]. The review also found that only 10 % of government and charitable investment in biomedical research in the UK was for treatment evaluation while over two thirds was directed towards basic research. The authors reasoned that waste is largely preventable if:

- Existing research is reviewed and analysed using high quality methods prior to funding more research, and
- Research questions are designed to be of relevance to patients and their clinicians.

### Bias in design

Good medical research begins with asking research questions responsive to the needs of patients and set in the context of existing research. In their book ‘Testing Treatments: Better Research for Better Healthcare’, the authors say that most of the global funding of medical research goes into laboratory and animal studies rather than research that is likely to have more relevance to patients [19]. In 2004 the James Lind Alliance (JLA) was formed with the aim of addressing this issue by generating research questions in favour of patient priorities and interests over those of researchers and industry [20].

It would be expected that animal studies are always carried out for sound scientific reasons but prestige, economy and convenience may influence decisions [21]. Publication in high impact factor journals is how scientists are now rated, and in a recent survey that looked at the convergence and divergence among clinical and basic scientists the authors found that basic scientists lacked involvement in the outcome of their research and how it translated to patients. Personal motivations came from scientific discovery rather than application, and the main interest was studying biological mechanisms in favour of research with little financial gain [22].

A large part of medical research is narrowly focused on drug development utilising the animal model in cellular and molecular biology and genetic research [23]. The discovery of how to genetically modify mice has produced a high percent of research using genetically altered (GA) (usually rodent) animal models. Laboratory mice are used in animal studies disproportionately more (95 % according to the US Foundation for Biomedical Research) than any other animal species. This may be more for convenience than scientific consideration but is now regarded as the norm. Mice are small, docile, easily handled, housed and maintained and they adapt quickly to new surroundings. Their reproduction rate is fast and lifespan brief, allowing for generations of mice to be studied in a short time-frame.

The research portfolio of the US National Institute of Health (NIH) is a notable example of how research priorities are weighted towards molecular and cellular biology and gene research based on animal studies [24]. But although it is known that patients respond to drugs differently according to genetic make-up, and that this can be a reason why drugs fail in some patients and not in others, pharmacogenomic animal models are causing difficulties with establishing appropriate predictive models for the research [25] which has not been without harm to patients and waste of research [26, 27].

Patients have their own treatment choices and preferences, which may not always include medication [28]. For instance, the refugee and immigrant sections of the population are high users of ethnic and traditional non-drug interventions and the JLA have found that some patients prefer coping strategies, nutrition, physiotherapy, surgery, complementary medicine and other under-researched options rather than drug therapy [20] and the Cochrane Depression, Anxiety and Neurosis Review Group found that exercise for depression is of equal efficacy to that of medication [29], and yet little funding goes into researching this intervention compared with funding drugs for depression.

Recommendation (1) RESPOND to patients' needs by asking research questions of relevance to patients and their priorities.

### Bias from irreproducibility and research misconduct

The attrition rate in drug discovery is reflected in the many failures of human trials at clinical research Phases II and III [14, 30]. This loss suggests that pre-clinical animal trials may not always have the necessary capacity to forecast the human response to drug therapy and toxicological studies. The Director of the NIH cautions that a high percentage of animal research may not be relevant to the human situation (Collins) [31], and it is estimated that 80-85 % of drugs effective in mice are ineffective in humans and more than 30 % of drugs passing animal tests for safety show toxicity in human trials (Geerts) [31]. The average rate of translation for animal models of cancer is less than 8 % [32].

Pharmaceutical companies are under extreme pressure from shareholders to raise share value, and investors are becoming increasingly aware of the irreproducibility of animal studies. As a result the NIH recently announced an initiative [3] to improve the quality of preclinical research, prompted by concerns of investors and some sections of the scientific community. Early-stage biotech venture capitalists have known for years that animal research is unreliable, but it is only recently that they have been publicly involved in addressing the issue for their own interests [33, 34].

A survey on data reproducibility in cancer studies reported that some pre-clinical investigators knowingly use unreliable data resulting in flawed research, which they then pressure support staff to publish [4]. This author is informed by a researcher using animals that they would rather remain silent about the 'pervasive flaws' in their field of animal research than report them. (Personal communication undisclosed for protection of researcher). Other acts of misconduct take place where basic researchers collude with the pharmaceutical industry to market drugs by accepting 'gifts' in various forms, in return for favours such as acknowledging the sponsor in their reports, providing pre-publication reviews of articles traceable to the gift, ghost-writing and ghost-management of publications, reporting unwarranted efficacy of drugs, under-reporting unfavourable results and selectively presenting posters and abstracts at scientific meetings [35].

Toxicologists have continued for decades to ignore genetic variation when selecting animal models even though to do so leads to unreliable results [36] and using too few animals in the design of experiments can be as wasteful as using too many because the research becomes 'under-powered' and consequently invalid leading to unrepeatable work and avoidable repetition of research. It was only recently in 2012 that industry realised the full impact of irreproducibility when it conducted a study that found that 47 out of 53 cancer studies published in top tier journals could not be replicated and yet were published as 'landmark' results. The author of the study concluded, 'Although hundreds of thousands of research papers are published annually, too few clinical successes have been produced given the public investment of significant financial resources. We need a system that will facilitate a transparent discovery process that frequently and consistently leads to significant patient benefit' [37].

Recommendation (2) REPLICATE animal studies independently using scientific evidence-based methods and standards prior to publication. Address research misconduct and protect whistle-blowers.

### Bias from lack of registration

Registration of animal trials is as necessary as it is for clinical trials [38]. Reviewers need to monitor the claims made in the research proposal against the published results in the same way as clinical trials. This cannot be done unless the protocol and aims of the research are recorded beforehand. The first time it was noted that prospective registration of animal studies is not in practice was in the BMJ in 2002 [39], but in spite of repeated calls there are no registers. This failure leaves animal studies vulnerable to researchers changing the endpoints and details of the research after it has started and allows them not to publish unfavourable or negative results of their research. In 2013 the Centre for Reviews and Dissemination (CRD) gave a presentation ‘Registration of experimental studies and systematic reviews’ [40], which highlighted the principles of registration for both animal and human studies:

- Availability of evidence to inform health care decisions
- Avoidance of publication bias and selective reporting bias
- Requirement of the Declaration of Helsinki
- Avoidance of unnecessary duplication
- Identification of gaps in research
- Facilitation of recruitment
- Promotion of collaboration
- Early identification of potential problems

Recommendation (3) REGISTER all animal trials prospectively on an open global register.

### Bias from poor reporting

Reporting and accounting for details of an animal trial in a study protocol is essential in order to avoid publication bias, assist replication and justify the research. Methods of statistical analysis used, sample sizes, inclusion/exclusion criteria, methods of randomisation, blinding, gender, strain, species selection, and age of animals all need to be reported. Al-Shahi Salman and colleagues refer to systematic reviews (SRs) that have shown fewer than 2 % of animal studies explained the basis for their sample size calculations [41].

Variables frequently confound research and need to be identified, measured and controlled to avoid damaging the internal validity of the study. For example, research results have been skewed by noise, infection, infestations, microorganisms and pathogens [42–44] and environmental temperature affected the results of cancer research [45]. More recently it was found that human gender disparity heightened stress levels in laboratory rodents affecting their analgesic responses [46]. Another study using only males of the selected animal models confounded the research. See [Table 1.] for a more comprehensive list of variables.

**Table 1:** Confounding variables in the design and reporting of animal study protocols (list not exhaustive)

Ages of animals
Animals added to replace drop-outs
Bedding type/quantity
Blinding (i.e. blinding the investigators and staff to allocation)
Breeding
Cage position on shelving
Cage size/material
Circadian rythms
Cleaning (chemicals, methods and manual or automated cage washing)
Colony size
Co-morbidity
Dosing style and route
Enrichment methods and practices
Environmental (other)
Exercise (methods, equipment, timing, amount, locations)
Feeding (equipment, methods, timing, amount)
Feeds (type)
Frequency of intervention
Gender (male and/or female included)
Gender mix and family and community (ratios)
Genetic variation
Genotype
Handling
Husbandry techniques
Infection or infestation (not part of the research i.e. pin worm, fleas etc.)
Lighting
Litter size
Methods of analysis
Methods used to conceal allocation sequence
Methods used to generate allocation sequence
Model type (induced, spontaneous, transgenic, negative, orphan, or other)
Noise (background and foreground)
Nutritional deficiencies due to lack of normal living conditions
Odours (e.g. perfumes worn by technicians, laundry and cleaning products and medications)
Polypharmacy (whether accounted for to mimic target population - humans)
Randomisation of animals (details of methods used)
Recording methods (manual, electronic)
Research methods
Sample size estimation (whether computed and statistical method of computation)
Sampling of style and route
Sources of animals (e.g. same model may vary depending on supplier used)
Species
Staff changes and variation of routines
Staff numbers and attitudes
Statistics (full information on statistical methods used)
Strain
Substantiation (whether results substantiated under a range of conditions)
Surgical procedures
Temperature
Timing of induction of disease
Timing of intervention
Transportation methods
Unit of analysis
Water and watering methods
Weight of animals

Several guidelines have been issued recently to improve poor reporting. In 2010 the ‘Animal Research: Reporting of In Vivo Experiments’ (ARRIVE) [47] was published, and then in 2011 the Gold Standard Publication Checklist (GSPC) improved and further elucidated the ARRIVE reporting requirements [48]. Following these, in 2013, Nature Journal  issued it's  own checklist to encourage authors to report details of their research precisely and methodically with more statistical information, setting higher standards for the research they publish and which will influence other journals to do the same.

The ARRIVE guidelines have been endorsed by over 300 major journals since they were first published, however, there has been some criticism that they are not adhered to [49], that they do not go far enough, and that animal trials should be carried out to the same standards as those set for clinical trials with greater attention paid to reducing bias [50]. Although the guidelines list suggestions for improved reporting, there is currently no requirement to comprehensively and uniformly identify and report all details of research in a study protocol. As a consequence there is a noticeable lack of consistency of reporting in journals making it difficult for reviewers and other readers of research papers to make fair assessments.

Recommendation (4) REPORT all details of the research in a well written protocol (plan of the research). Journals to improve on and enforce adherence to guidelines for animal research.

### Bias from non-publication

A survey on publication bias in laboratory animal research found that researchers in academia estimated only 50 % of animal research is published while researchers in industry estimated only 10 % is published [51]. In 2010 a report by the Health Technology Assessment (HTA), looking at publication and related biases in medical research [52], concluded that publication bias could be one of several explanations for the lack of translation from basic research to the clinical situation. In animal studies of neurological disease, for instance, reviewers found excess significance bias and summarised that only eight of the 160 evaluated treatments should have been tested in humans [53].

In June 2013 Doshi and colleagues published a proposal for restoring invisible and abandoned clinical trials. The project known as RIAT (Restoring Invisible and Abandoned Trials) aims to publish or re-publish all clinical 'grey literature', but the same improvement should be applied to animal studies so that reviewers can trace the research back to the original research, to get a complete picture of all the results and processes that have led to the clinical studies.

Recommendation (5) RECORD (publish) the results of all animal trials in science journals regardless of whether the outcomes are positive, negative or null.

### Bias from lack of systematic review

It is estimated that there are over 7 million published animal studies of which over 5 million are on PubMed [54] and yet only 91 SRs of animal studies were published during the years 2005 to 2012 inclusive (on any topic). In only 48 of the SR's was a risk of bias or quality assessment performed, a necessary step in SR methodology. The quality of the primary studies was low, and of these only approximately 20 % checked several issues for the risk of bias and internal and external validity [55].

In 2002 a SR in the BMJ on fluid resuscitation was perhaps the first paper to raise the question of why SRs of animal studies were not prevalent [39], and in the same year the Lancet published a paper which called for the requirement of SRs of all relevant animal and human studies before proceeding with clinical trials [56].

The Nuffield Council on Bioethics produced a report in 2005 which directed a recommendation to the leading funders of animal research, the pharmaceutical industry and animal protection groups in the UK to fund SRs of animal studies "to evaluate more fully the predictability and transferability of animal models" [57]. Following that in 2007 the HTA commissioned a pilot study to assess the extent of concordance between animal and human studies of treatments and determine whether SRs of animal trials would help to answer questions about their relevance to human treatments and interventions. The study  found some discordance between the animal and human studies and confirmed that SRs are valuable, but it also found insufficient evidence to quantitatively determine the generalisability of animal research to human medicine and recommended that funding should go into this area of research [58].

There has been a small increase in the number of SRs of animal trials since these reports, but they remain scarce in comparison to those of clinical trials [59] and almost non-existent in comparison to the amount of published animal literature.

The study of animal models of stroke is probably the most reviewed field of animal studies for relevance to clinical research, beginning in 2001 when Horn and colleagues were arguably the first researchers to conduct a SR of animal studies. Their review of nimodipine [5] found the methodological quality of the animal studies was poor, and that only 50 % of the studies were in favour of the intervention with no convincing evidence to start clinical trials which had involved thousands of patients. The animal trials of nimodipine continued for over 15 years after clinical trials had started, and some animal and clinical trials ran concurrently, which the reviewers found surprising. Other SRs of stroke research found bias and methodological weaknesses, and that almost no new drug interventions translated to the clinic following decades of animal and clinical trials [60].

Researchers are more likely to receive funding to publish new animal studies and develop more animal models [19, 61] than to critically appraise existing studies and models. Even while debate is underway amongst researchers about how or whether to take animal data to the clinic, the clinical trials may still be carried out [32]. Participants in Phase I trials should be adequately informed before signing consent forms, [62] and if SRs have not been conducted, or are not available, the advice is not to enter the trial [19].

Recommendation (6) REVIEW animal trials using systematic review.

### Bias in the regulation of animal research

In an article in Nature in 2004, calling for a more evidence-based regulatory system in the drug industry, the author was critical of the existing system saying that many regulations are based on expert opinion rather than evidence and that there needs to be quantitative assessment of the predictive value of preclinical studies [63]. A decade later, in an article in Drug Discovery Today, an industry-based researcher (preclinical and clinical) commented that the regulatory agencies require preclinical investigational new drug packages (applications) to contain efficacy and other data from studies that use animal models even where they are known to be unpredictive [64].

The regulation of animal research in the UK is guided by three principles known as the 3Rs (refine, reduce and/or replace experiments using animals), with the intention of contributing to the welfare of laboratory animals. They were first published in England by animal researchers Russell and Burch in 1959, a time when animals were mainly used for screening chemicals for biological activity [65].

The 3Rs rest on the assertion that research questions about humans can be answered by studying animals. The first two principles, 'refinement' and 'reduction', fully support this notion and have little bearing on scientific questioning of animal models. The third principle, known as 'replace', supports the notion whereby animal models are regarded as the 'gold standard, and all non-animal research models are classified as 'alternatives', which must be validated against animal models before they can 'replace' the animal model in practice. Although there is little agreement between companies and academia about the methods of validation of animal models, the non-animal technologies are generally regarded as inferior by regulators even where researchers develop effective human-based models that are highly predictive [64, 66].

The 'harm-benefit assessment' (harm to laboratory animals in terms of welfare – benefit in terms of human health) is the earliest part of the approval process of animal research. This assessment is carried out in the UK by the Animals in Science Regulation Unit (ASRU), a body of twenty three inspectors, mainly veterinarians. They inspect the 180 research establishments throughout the country to see that they comply with animal welfare requirements [67]. Their remaining time involves assessing and authorising each animal research application using the 'harm-benefit assessment'. However, the assessment is neither open nor transparent and relies on the individual expert opinion of the inspector.

Details of how many applications inspectors approve or turn down and the reasons why are not disclosed, but it has been reported that an application may be turned down by one inspector in one part of the country while the same project is approved in another [68]. From the little information available about the work of inspectors and how it is regulated, it would appear that the 'harm-benefit assessment' is mainly concerned with assessing animal welfare and compliance with the 3Rs.

Definition of the 'benefits' part of the analysis is quite generalised in the guidelines [69], and there is no requirement to carry out a SR before making the application. Instead, a set of general questions is asked such as "Clarify the current state of knowledge on which your project intends to build" and "Is the work scientifically sound?" The inspectors rely on the accuracy and honesty of the answers given by the applicant on the forms.

Once an inspector has approved a project licence application, it is passed to a local animal ethics committee known as an Animal Welfare Ethical Review Board (AWERB). Members are selected from the institution where the research is taking place, often including the researchers themselves. Concerns have been raised about this practice and that animal researchers and AWERB members are not usually trained in research methodology and statistical analysis [70]. Some Universities actively reject potential panel members with appropriate expertise and less subjective involvement (personal communication undisclosed for protection of reviewer). This is quite common and may affect the legitimacy of the approval process [71, 72].

Like the ASRU, the purpose of the AWERB is to weigh the harm-benefits of the research project. However, like the assessments of the ASRU, the review process is not transparent or open to inspection and consequently there is no connection between the approval of animal research and the published results. The expertise and aims of both the ASRU and the AWERB are orientated towards animal welfare compliance, and while ethical reviews will contain detailed information about animal procedures pertaining to the 3Rs there is limited scientific information. Since there is no publication of the review it is impossible to monitor its effectiveness. A group of European researchers studying the ethical review process [73] says that this could be rectified by the following actions:

- Journals to require the complete legal reference document including the name of the ethical entity that gave authorisation.
- Make the ethical review approval records available (without disclosing individual researchers' identities or personal details) either by publication as online supplementary material in journals or on a separate database.
- Prospectively register all animal trials with an identifying number.

There is mounting criticism in the US that the committees equivalent to AWERBs, known as the Institutional Animal Care and Use Committees (IACUCs), approve nearly every animal project that they review. As a consequence physician-scientists are turning away from human-based research in favour of animal research for which they can expect ease of approval and funding. In contrast, the US Institutional Review Board (IRB) that reviews clinical projects is criticised for creating numerous hurdles in the approval process, increasing the trend for physician-scientists to turn towards animal research [74].

A Social Science and Medicine study in 2010 on the use of rodent and GM mice models of carcinogenic risk assessment revealed how industry drives the science underpinning safety testing of drugs by manipulation of the validation of animal models for its own ends, and how the regulatory bodies collude with industry until there is a crisis and then the regulations are tightened only to protect the reputation of the regulators. Afterwards regulation drifts back to accommodate industrial motives [75]. The study raises questions about the credibility of the validation of animal models, how profit-making influences animal testing and how industry is deeply embedded in the regulatory process.

Recommendation (7) REGULATE animal research using evidence-based methods and overhaul the regulatory system.

### Bias in the hypothesis and quantitative appraisal of animal research

The latest student guidance notes on the selection of animal models state 'the rationale behind extrapolating results to other species is based on the extensive homology and evolutionary similarity between morphological structures and physiological processes among different animal species and between animals and humans'. But despite decades of research relying on this rationale, expounded by Calabrese in 1983 [76], the hypothesis has not been verified [77].

The validation process raises questions about whether animal models can be regarded as a reliable scientific tool [78]. Non-animal technologies undergo rigorous and lengthy (years) testing against a 'gold standard' animal model before they are approved. Even when it is completed the adoption of the new model or method may be overridden by the regulatory bodies, and non-predictive or irrelevant animal models used [64, 66] But animal models have not undergone validation to the same extent or to the same exacting standards [79]. Where it is carried out, validation is limited and uses disparate methods of assessment. The inaccessibility of raw animal and clinical data, lack of consensus on development and validation of animal models, and no common agreement of testing methods between companies and academia, can result in an inconsistent and flawed statistical and analytical validation [80–84].

Bennani lists several human diseases for which animal research models are non-predictive of clinical outcome:

- Oncology
- Immunology
- Psychiatry
- Central nervous system (CNS)
- Other neurological (e.g. pain)
- HIV
- Hepatitis C (HCV) infections

For some other conditions he says animal models are 'more reliable' such as influenza, bacterial and fungal infections, measuring CVD and LDL and simple blood chemistry read-outs such as glucose or cholesterol [64]. However, animals rarely characterise a human condition exactly. No animal models of influenza, for instance, mimic human influenza and researchers model for the virus in several different species in the hope that gathering a collection of data may be useful in drug discovery [85, 86].

A workshop on animal models of CNS reported in 2013 that there are no animal models that predict human diseases or disorders [87]. The consensus was that researchers could probably only hope to model disease mechanisms or phenotypes, and that animal models should not be called models of a particular human condition or disease but instead labelled for the hypotheses or mechanisms studied.

In a recent paper detailing how to use meta-analysis as a solution to translational problems in animal research, the authors claim that animal studies are not only 'crucial' to understanding disease mechanisms but also for testing drugs for toxicity and efficacy [88]. They say that success in translating findings from animal studies to humans depends on understanding sources of heterogeneity in the published studies. While their paper is a useful and practical guide to systematically analysing animal data from the study of disease mechanisms in animals, their assertion that the use of animal studies is 'crucial' for testing drugs for toxicity and efficacy is challenged by evidence from academia [59], industry [64, 89] and toxicology [90] and overlooks four problems as discussed in a paper on extrapolation and translation of research [91]. The problems are summarised as follows:

- Our understanding of mechanisms is often incomplete.
- Knowledge of mechanisms is not always applicable outside the tightly controlled laboratory conditions in which it is gained.
- Mechanisms can behave paradoxically.
- Mechanistic knowledge faces the problem of the 'extrapolator's circle'.^2^

The authors warn that studying mechanisms in the study population (animals) in order to translate the finding to the target population (humans) is only valid in exceptional cases and that we need more reliable solutions.

The response to address difficulties with the translation of animal experiments to the clinic has mainly been 'build better animal models', and increasing numbers are produced. JAXmice® alone stocks tens of thousands of types of strains of mouse models to choose from [92], and attempts at creating better models have resulted in what the industry labels 'humanised' mice models (mice carrying human genes, cells, tissues and/or organs), animals with 'sterile' immune systems, transgenic animal models (Tg) and chimeric models (animals engineered with human material inserted), such as the NOD.Cg-Rag1tm1Mom Il2rgtm1Wjl Tg(HLADRA,HLA-DRB1*0401)39-2Kito/ScasJ strain [93].

For a scientific method of testing to be valid, it should possess what is termed 'predictive value' with 'demonstrable evidential weight'. A detailed discussion on the predictive value of animal tests can be found in the Journal of the Royal Society of Medicine [94], but it is only recently that a thorough statistical evaluation of toxicological and pharmacokinetic animal data has been carried out. This was made possible by the (unusual) cooperation from industry when it released a large canine dataset. The analysis found no evidential weight to support canine data to predict efficacy and toxicology of medical compounds in clinical trials [80].

In fields of scientific research such as aeronautics, space science, computer science or engineering, it is generally accepted as important to revisit failed projects, reappraise the studies and learn from mistakes. A lack of predictive value in a research model would be acknowledged as a failure of the model. But although a significant amount of animal research does not produce hoped-for results, little is critically appraised by SR and meta-analysis [95]. This wasteful approach of abandoning unsuccessful research and starting again without re-evaluating the failed work would be considered inadmissible in most science-based industries.

A study in 2012 by van Meer and colleagues found that animal data were not predictive for detecting serious post-marketing adverse drug reactions in patients and should not be included in prospective pharmacovigilance studies [77]. Instead, the researchers have developed a method which, they say, can be adapted to evaluate all drugs on the market as well as those still in development to assess the predictive value of animal studies. They summarise the situation and what needs to be done:

- 'The adoption of the precautionary principle by the regulatory authorities and the relative ease with which this burden of proof is accepted by the pharmaceutical industry - without attempts to improve the current paradigm - has created a stalemate in which animal studies, predictive or not, continue to exist with little room for innovation. Stakeholders in industry, academia and regulatory agencies, need to critically assess animal studies and discuss their predictive value in all earnestness and with the scientific facts at hand. From this, possibilities based on scientific facts may develop which allow new technologies to be implemented that predict the safety and efficacy of therapeutics equal to or better than animal studies do. A way forward would be for the pharmaceutical industry to share clinical and laboratory data generated at all stages of product development with collaborating stakeholders, to enable a complete and transparent analysis of the predictive value of animal studies for drug development' [96].

Recommendation (8) REAPPRAISE the validity of the hypothesis that animals make reliable models for humans and that they possess predictive value.

### Bias from lack of economic assessment

The production of animal models is a lucrative and competitive business. It has been estimated that the UK alone expended 4.02 million animal models in research in 2013, of which over 3 million were mice. The mouse model (mentioned above) costs $155.35 (£94) per mouse at weaning age including shipping [97]. Some mouse models, such as the AD (Alzheimer's Disease) mice cost more, for example, the beta-amyloid transgenic mouse comes in at over $500 (£303) each. The Jackson Laboratory Humanized CD34+ NSG mice are as much as $1300 (£760) per mouse.

Expensive price tags on animal models and associated expenses indicate that animal research is a costly component in the drug discovery process, and which is further increased by high levels of customisation according to the field studied. Animal models can be purchased off-the-peg or customised from online stores where they are sold on an industrial scale [98, 99], as are specialised animal feeds and associated technical equipment [98, 100]. For a list of potential cost factors in animal research see [Table 2].

**Table 2:** Cost factors in animal research (list not exhaustive)

ACU/OCU staff members salaries
Administration and IT
Air filtration systems in the vivarium buildings
Anaesthetics
Analgesics
Animal health diagnostic services and equipment
Animal models (various)
Animal transfer stations (change stations)
Autoclaves
Automated cage washing equipment
Bedding (specialised types of bedding)
Bedding disposal stations and units
Biological work stations Blood analysis equipment
Bottle fill stations
Breeding imports and exports
Cage card holders (various for different size and types of cage)
Cage shelving
Caging and cage trolleys (various for different sizes and types of species)
Cleaning and scrub equipment and products (general)
Cryostat equipment
Data collection equipment
Decapitators
Diagnostic and monitoring equipment
Dismembrators
Energy costs
Enrichment products
Feeding equipment ( various sizes for different sizes of animals and species types)
Feeds (specialised)
Forklifts
Handling gloves and clothing (specialised according to species)
Heating and lighting equipment
High-efficiency particulate air filtration systems
Housing (specialised premises with airlock barriers between vivarium rooms)
Husbandry facilities and equipment
Imaging Xray and CAT
Importation & Exportation costs
Incubators
Inhalation equipment
Intensive care units (ICU)
Lab technician specialised workwear
Laryngoscope
Load carts
Microscopy
Monitoring equipment
Necropsy tables (various sizes)
Plethysmometer
Power lift
Protective clothing for automated cage cleaning
Respirators
Restraint frames and jackets (customised according to species)
Sanitation and garbage collection (general and specialist)
Security staff salaries
Sinks and tables
Specialist feeds
Specific pathogen free cage housing rooms (in addition to regular ones)
Staff Training
Stationery and recording equipment
Sterile and germfree transportation equipment
Sterilisers
Surgical cleaning and scrub equipment and products
Surgical equipment (i.e. cauterisation, homeothermic, stereotaxic, lighting)
Surgical specialised workwear
Surgical, blood and crematory waste services and equipment
Syringe pumps
Transportation (International & Domestic)
Ultraviolet light-treated deionized water lines
Urine analysis
Urine and feaces ammonia levels monitoring equipment
Vacuum units
Ventilators
Watering equipment (different sizes and types for different species)
Water (for animal drinking, cage cleaning and vivarium and surgical cleaning)
Weighing and scales equipment (different sizes for different species)

In 2003 a report by the Health Economics Research Group (HERG) found that only 2 %-21 % of basic research translated into clinical benefit for patients [101]. The authors had examined a publication from 1976 in which it was claimed that 62 % of basic research articles were judged essential for later clinical advances [102], and as a direct result of that publication, funding has been directed in favour of basic research. Although it is now understood that applied clinical research has greater benefit for patients [103–105], proportional funding allocation has yet to accord with the evidence.

A regular and transparent account of spending on animal research does not appear to be carried out anywhere. However, in 2009 it was estimated that approximately £8 billion (€10b /US$14 billion) was spent on animal experimentation worldwide of which £1.7 billion (€2 billion/US$2.8 billion) was for toxicological studies [106]. In 2009/10 the Medical Research Council (MRC), a major funder of animal research in the UK, published that it spent 30 % (£227 m) of its annual research budget on research involving animal species.

DiMasi and colleagues have carried out several economic assessments to evaluate the costs of drug development and some included basic research costs. Their estimation of preclinical cost per approved new drug in 2000 was $335m, but they found that the way in which companies reported the costs of basic and animal research were so variable that it was not always possible to disaggregate the data usefully [107].

The Home Office Annual Statistics of Scientific Procedures on Living Animals is the closest form of monitoring of animal research in the UK, but the report does not document details of expenditure or funding of animal research. In 2012 it reported that 48 % of animal research was carried out by academia; 27 % by industry; 13 % by public bodies and 9 % by NGO's.

Recommendation (9) RATIONALISE animal research by carrying out a full economic assessment.

### Bias from reductionist-materialism in science

Researchers working with animal models understand that the resulting data cannot always be relied on for predictive value and translation to patients. They operate within a narrow remit embedded in reductionist-materialism, a belief in science which holds that everything can be reduced down to an observable and controllable material part and isolated from other neighbouring or related parts with little or no consequence [108, 109]. An example of how this can lead to problems is seen in gene research when it ignores the behaviour and effects of ‘cell intelligence;’ an oversight that produces misleading data. Findings in epigenetics explain that local and external environmental factors can influence and control the behaviour of genes and must be taken into account.

Since publication of the FDA White Paper in 2004 [11] there has been little progress in discovering scientifically sound predictive animal models, despite more than adequate funding and support for the development of new animal models [12, 13]. Nevertheless, there are pockets of innovation where researchers are developing tools and models that may reliably predict the human response:

- In the field of CNS drug discovery, Geerts and colleagues model disease using a 'humanised' approach with what they call 'computerised biophysically realistic disease-modelling' while incorporating the issue of polypharmacy in their methods - something that can never be achieved in animal models or human trials [110]. To perfect their in silico models they have made use of some existing pre-clinical published animal data (mostly primate) and combined it with human imaging and genotypic and clinical information. They also pay attention to quality of life and patient and care-giver needs when designing research, which they focus on patient-centred healthcare [111].
- At Columbia University Professor Raza has spent the last 30 years collecting human cells, biopsies and samples with which she has assembled a tissue repository supported by a databank of clinical, pathologic and morphologic data. The repository is used for research into myelodysplastic syndrome (MDS) but when Raza applied to the NIH for funding to validate her human-focused research, which she describes as "startlingly predictive," the response was that she should first reproduce the work in mice [66]. It took over three years to gain funding, and then only after some of the aims of the research were dropped to avoid unnecessary and inappropriate animal studies  (private communication with the author).
- Collaborative work by the Wyss Institute at Harvard University and AstraZeneca is researching innovative ways of predicting the human response with 'Biologically Inspired Engineering' to create human organ-on-a-chip technology [112], which they hope will in the long-term fulfil the search for predictive models for humans. And transition is in progress in toxicology testing at the National Center for Advancing Translational Sciences (NCATS), where they are developing evidence-based toxicity test methods [106].
- A group of oncology surgeons writing in the American Journal of Translational Research earlier this year proposed a number of different methods for improving the translation of oncology research to patients such as Phase 0 patient trials (micro-dosing), epidemiological studies, autopsies, in vitro studies, in silico computer modelling, silicon and plastic chip technology, and microfluidic chips [32].

Recommendation (10) REINVENT and support new ways of looking at pre-clinical research.

### Bias in debate and presentation of animal research

Animal models are promoted by industry as a commodity for drug development, rather than the complex biological systems that they are. Companies overstate their value with inflated, obtuse or scientifically unsound advertisements for commercial gain. Jackson Laboratories, for example, claim 'Humanised mouse models represent powerful tools for studying haematopoiesis, inflammatory disease, viral host-pathogen interactions, and are helping to accelerate the development of novel therapies in HIV infection and oncology'. JAXmice®, states, 'Because mice and humans share 95 % of their genes, mice are an effective and efficient model for human diseases'. Such statements may be misleading when advertising animal models with claims that have not been verified [113]. Mouse and other animal models may well be involved in many fields of research, but evidence is still needed to verify their predictive value for humans.

Investors in biotechnology will be aware that pharmaceutical companies usually issue a ‘Safe Harbor’ declaration at the end of their press releases. These refer to what are termed ‘Forward-Looking Statements’ which contain claims about the predictive value and subsequent monetary value of work carried out. In effect, the statements provide legal cover for risks and uncertainties that may arise from investment in companies that make drugs, medical products or devices to be tested in animal and clinical trials and then released onto the market. These statements indicate how human disease is set in the context of commercial enterprise, with emphasis on financial reward from sales of drugs.

University and research institutions have been found issuing inflated claims about their own research results in order to attract maximum media coverage. Press releases frequently contain unsubstantiated declarations about ‘medical breakthroughs’ and ‘positive results' but without explaining that the findings may be in the early pre-clinical animal research phase and that have yet to be verified [114].

Recommendation (+) REPRESENT animal research honestly and with scientific evidence.

## Strategy

To support the mission of better science for better healthcare, the charity SABRE Research UK suggests a strategy for improving pre-clinical research:

1. Systematic review: a moratorium on animal trials (in line with calls for a moratorium on efficacy clinical trials) while a large-scale global programme of systematic reviews of existing animal studies is carried out, including systematic reviews of the value of animal research for the seven leading causes of death (Western world).
2. Scientific evaluation: a) evaluation of the validation methods of animal models and in relation to the higher standards set for the validation methods of non-animal models and technologies. b) a large-scale research programme to evaluate the predictive value of existing animal studies (as set out by van Meer and colleagues).
3. Inquiry: a full scale inquiry into why unreliable animal data are accepted by regulatory authorities while at the same time non-animal models and technologies with predictive value are less likely to be adopted.
4. Audit: a full and independent public health audit of spending on animal research.
5. Apply 10Rs + Recommendations^3^ [See Additional file 1]

## Conclusions

The US pharmaceutical industry takes an average of 15 years to achieve marketing approval for a new drug [115] and the cost of investment in developing a new medicine is now estimated at $1.3 billion [116]. Given that drug development is an expensive and lengthy process, with many compounds failing for each one that succeeds and that prescription drugs rank third in the leading causes of death in the Western world [117], it will be valuable to conduct an audit to assess the costs and benefits of animal research and its sustainability.

The proportion of published animal research that has not been systematically reviewed is hard to quantify, but the high volume of research output means lack of critical appraisal is wasteful particularly when funding goes into research that results in more publications about the limitations of animal models [118]. The way animal research is published tends to spawn new research before effectively synthesising existing research. Researchers identify numerous difficulties that emerge during attempts at translating animal data to the clinic [119] and then assert that 'further research is needed', without first reviewing what went wrong. This paves the way for the same researchers and others in the field to produce more papers on similar, or in some cases virtually identical research, without adequate review. There is little encouragement by funding agencies to critically appraise research, and little incentive for researchers to consider that their research path may be unproductive and new ways of looking at questions might be necessary.

Some clinical researchers are now calling for a return to real evidence-based medicine in order that a highly individualised system of healthcare can be delivered to fulfil the long-awaited promise of personalised medicine. They say that there is too much research being produced, and which has overtaken the time needed to evaluate it. Less but better quality human-centred research is the message [120].

A proposal to suspend clinical research and appraise all existing data has recently been suggested [109], but a moratorium with a full and extensive appraisal of existing animal research may be more important given that so few animal studies have been systematically reviewed. It could be time to stall the output of animal research and systematically review existing literature to see what can be learnt before proceeding to carry out more research. The work by Geerts and colleagues demonstrates this can be a productive approach [110, 111] and it may help the pharmaceutical industry and academia to learn from drug failures and avoid repeating the same mistakes.

The almost limitless repository of heterogeneous animal studies makes it possible to selectively cite papers that will support almost any claim made about animal research. It is important, therefore, that clinical investigators accept responsibility for ensuring that the animal data they use is scientifically sound before proceeding with human trials.

Research to determine whether negative results of animal studies may lead to the abandonment of research that would be effective for patients needs prioritisation. SR and scientific evaluation of the validation methods of animal models is also needed, so that robust and concordant methods can be developed, and complete and transparent analysis of the predictive value of animal studies should be undertaken without further delay.

The problem of how bias in animal research can falsify medical research may not reach the understanding of a wider audience. Many journals remain closed-access, with valuable content locked behind paywalls when patients, medical students, healthcare workers, researchers, libraries, policy makers and journalists need access to medical literature to make informed decisions. [121, 122] While debate about animal research and whether it is relevant to human health continues to lack quality [123], the problem remains that allowing bias in the research to go unchallenged is unethical for patients and research participants. If voluntary commitment to apply the proposed measures cannot be relied on, action may need to be taken by the public before real improvements are made.

### Declarations

There is a serious issue stemming from flawed animal research, which needs to be addressed in the interests of human health and safety. It is not the purpose of this article to discuss animal welfare or moral issues arising from the use of animals in research which are well documented elsewhere but that any future debate or decisions about animal research are informed by better quality and more reliable scientific evidence.

## Endnotes

^1^The noun 'data' in the title is to be understood as the mass noun, not the count noun.

^2^For discussion on the ‘extrapolators circle’ please refer to Darden L., Craver C. Strategies in interfieled discovery of the mechanism of protein synthesis. *Stud Hist Philos Biol Biomed Sci*. 2002;33:1-28 and Glenman S. Modeling mechanisms. *Stud Hist Philos Biol Biomed Sci.* 2005;36:443-464.

^3^The 10Rs + Recommendations was circulated in December 2013 to animal researchers and systematic reviewers and at http://sabreresearchuk.wordpress.com/ for invited comments and which were taken into account in the final draft. Revisions of the 10Rs + have been made up to the date of this publication.

## Additional file

Additional file 1: The 10Rs+ Recommendations Table. ᅟ
