# Supplementary material for: Can animal data translate to innovations necessary for a new era of patient-centred and individualised healthcare? Bias in preclinical animal research
Source: BMC Med Ethics. 2015 Jul 28;16:53. doi: 10.1186/s12910-015-0043-7 (PMC4517563; doi:10.1186/s12910-015-0043-7)
Supplement: Additional file 1: The 10Rs+ Recommendations Table. — ᅟ [file 12910_2015_43_MOESM1_ESM.docx]

| 10Rs+ (Updated: 20/07/2015) |  | DEFINED | **WHAT THIS MEANS** |
| --- | --- | --- | --- |
| **RESPOND** | 1 | Respond to patients' needs. | Ask research questions of relevance to patients' and their priorities. Engage healthcare consumers (patients) in research that is of interest and importance to them. |
| **REPLICATE** | 2 | Replicate animal studies independently. Protect whistleblowers. | Reproduce the animal study elsewhere independently to determine whether it is repeatable. Safeguard the integrity of research by protecting whistleblowers. |
| **REGISTER** | 3 | Register all animal trials prospectively. | Create an open global register so that animal studies are registered beforehand. Research is then traceable and reviewers can access all relevant information. |
| **REPORT** | 4 | Report the details of animal research. | A plan of the research must include *all* relevant details for replication and transparency and so that reviewers can appraise the research more reliably. |
| **RECORD** | 5 | Record (publish) the results of all animal trials. | Publish results of *all* animal trials whether positive, negative or null to avoid publication bias and so that reviewers can appraise the research more reliably. |
| **REVIEW** | 6 | Review animal trials using systematic review. | Carry out a comprehensive programme of systematic reviews before funding is granted for more animal trials. Carry out a systematic review of relevant animal and human trials before conducting any clinical trial that relies on animal data. |
| **REGULATE** | 7 | Regulate animal research and overhaul the regulatory system. | Improve the regulatory system by using evidence-based methods throughout all stages of regulation. Make available the documentation of the ethical review process and legal documents. |
| **REAPPRAISE** | 8 | Reappraise the validity of the hypothesis that animals make reliable models for humans. | Reappraise animal research and animal models using systematic review and other evidence-based methods to assess the value of animal research and animal models. Determine the 'predictive value' of animal research. |
| **RATIONALISE** | 9 | Rationalise animal research by carrying out a full economic assessment. | Document all spending on animal research and make it open and accountable to the public, patients, healthcare workers, funders and policy makers. |
| **REINVENT** | 10 | Reinvent and support new ways of looking at pre-clinical research. | Balance reductionist-materialism in science with innovative thinking, open science, and collaboration to help overcome obstacles in problem solving. |
| **REPRESENT** | + | Animal research to be represented honestly and transparently. | Debate and represent animal research honestly, openly and reliably in journals, press releases and the media supported by evidence-based material and references. |
